# Inflammation‐induced macrophage lysyl oxidase in adipose stiffening and dysfunction in obesity

**DOI:** 10.1002/ctm2.543

**Published:** 2021-09-16

**Authors:** An Huang, Yi‐Shiuan Lin, Ling‐Zhen Kao, Yu‐Wei Chiou, Gang‐Hui Lee, Hsi‐Hui Lin, Chih‐Hsing Wu, Chin‐Sung Chang, Kuo‐Ting Lee, Yuan‐Yu Hsueh, Pei‐Jane Tsai, Ming‐Jer Tang, Yau‐Sheng Tsai

**Affiliations:** ^1^ Institute of Clinical Medicine, College of Medicine National Cheng Kung University Tainan Taiwan Republic of China; ^2^ Department of Physiology, College of Medicine National Cheng Kung University Tainan Taiwan Republic of China; ^3^ International Center for Wound Repair & Regeneration National Cheng Kung University Tainan Taiwan Republic of China; ^4^ Department of Medical Laboratory Science and Biotechnology, College of Medicine National Cheng Kung University Tainan Taiwan Republic of China; ^5^ Department of Family Medicine National Cheng Kung University Hospital Tainan Taiwan Republic of China; ^6^ Department of Surgery National Cheng Kung University Hospital Tainan Taiwan Republic of China; ^7^ Division of Plastic and Reconstructive Surgery, Department of Surgery National Cheng Kung University Hospital Tainan Taiwan Republic of China; ^8^ Center for Clinical Medicine Research National Cheng Kung University Hospital Tainan Taiwan Republic of China

Dear Editor,

Obesity is associated with adipose tissue (AT) fibrosis with aggregation or crosslinking of collagen fibers.^1^, ^2^ Lysyl oxidase (LOX) gives rise to collagen crosslinking, while upregulated LOX is reported in AT of obese humans and mice.^3^, ^4^ Our data showed higher second harmonic generation signals as well as collagen crosslinks in *ob/ob* AT than wild‐type (WT) AT (Figure 1A and B). In *ob/ob* AT, expression of *LOX* was highly upregulated among the LOX family, while a similar trend of LOX increment was also found in high‐fat diet‐induced obese mice (Figure 1C and S1). The *ob/ob* AT also exhibited higher LOX protein levels and enzymatic activity (Figure 1D and E). Increased AT stiffness was previously found in obese subjects non‐invasively.^5^ Our direct measurements by atomic force microscopy (AFM) showed a similar trend by yielding higher effective Young's modulus (E_eff_) in *ob/ob* mice and obese human subcutaneous AT (Figure 1F and G). With adipose tissue derived from obese subjects before and after weight loss surgery, we observed significantly attenuated *LOX* expression but not that of other *LOX* family members (Figure 1H), accompanied by reduced stiffness (Figure 1I). While obesity‐induced physical property changes in AT may confine adipocyte functions,^6^ the origin and consequences of structural changes in AT as well as the augmented LOX during obesity await investigation.

**FIGURE 1:**
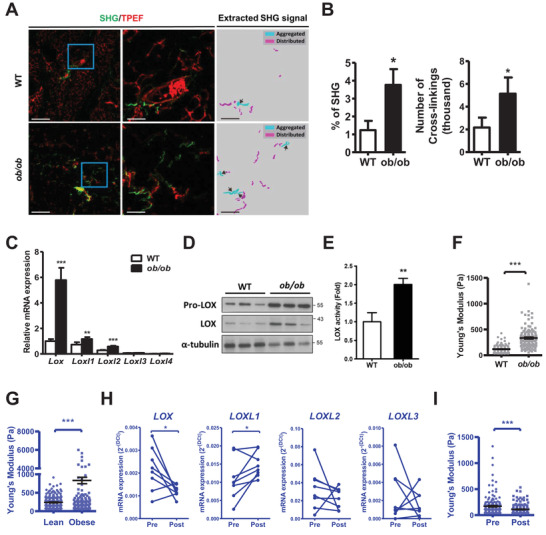
Obesity is associated with increased collagen crosslinking, LOX, and stiffening in the AT. (A) Overlaid images of second harmonic generation (SHG) and two‐photon‐excited fluorescence (TPEF) and extracted SHG signals of gonadal AT from 10‐week‐old WT and *ob/ob* male mice. Magnification of the blue square in the left panels is shown in the middle panels, which is then transformed into extracted SHG signals in the right panels. (B) Quantification of SHG percentage and number of crosslinks (intersections) accessed from signals in (A). (C) LOX family gene expression in gonadal AT of 10∼12 week‐old male WT and *ob/ob* mice. mRNA levels (*n *= 4∼5) are expressed relative to average expression of *Lox* in WT mice. (D) Immunoblot analysis of pro‐LOX and LOX and (E) LOX enzymatic activity in the gonadal AT of 10∼12 week‐old male WT and *ob/ob* mice. (F) Effective Young's modulus (E_eff_) of 10‐week‐old male WT and *ob/ob* gonadal AT by AFM. (G) E_eff_ of the subcutaneous AT from 3 lean and obese subjects. (H) LOX family gene expression in subcutaneous AT of eight obese subjects before (Pre) and 6‐months after (Post) weight loss surgery. (I) E_eff_ of human subcutaneous AT obtained from three subjects before and 6‐month after weight loss surgery. *P < 0.05, **P < 0.01, and ***P < 0.001 by Student's *t*‐test in (B, C, and E); by paired Student's *t*‐test in (H); and by Wilcoxon signed‐rank test in (F, G and I). Scale bars in the left panels of (A) are 200 μm and in the middle and right panels of (A) are 50 μm. Sample numbers and measurements taken in (F, G and I) are shown in Table S1. Blue color in (G, H, and I) indicates the results from humans

Immunofluorescence staining revealed increased LOX signals in *ob/ob* AT, predominantly expressed in crown‐like structures and was highly associated with F4/80 signals (Figure 2A and B). To test whether obesity upregulated LOX in macrophages, we isolated peritoneal macrophages from WT and *ob/ob* mice and found that mRNA and protein levels and enzymatic activity of LOX were significantly augmented in *ob/ob* peritoneal macrophages (Figure 2C–E). Moreover, *ob/ob* peritoneal macrophages showed the ability to stiffen type I collagen gel, a mimicry of AT environment with its predominant extracellular matrix protein (Figure 2F). Conversely, our data demonstrated a reduction in *LOX* transcript levels in peripheral blood mononuclear cells (PBMCs) from human subjects 6 months after weight loss surgery, which was further reduced in PBMCs from subjects 12 months after surgery (Figure 2G). These results implied obesity was associated with increased macrophage LOX.

**FIGURE 2:**
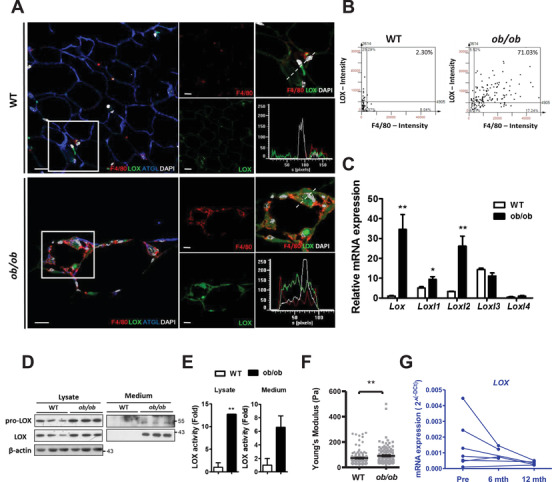
Obesity up‐regulated the crosslinking mediator, LOX, in macrophages. (A) Confocal image for co‐staining of LOX (*green*), F4/80 (*red*), ATGL (*blue*), and DAPI (*white*). The enlarged images of the squared area in the left panel are presented in the right panels, where the individual channels are shown in *red* (F4/80), *green* (LOX), and *white* (DAPI). The fluorescence intensity of the individual channel in the right bottom panel represents the dot line cross‐sectioning the image in the right upper panel. Scale bars are 20 μm. (B) The scattering plot from semi‐automated analysis of the fluorescent image shows an association between LOX and F4/80 in the gonadal AT of 10∼12 week‐old male mice (2.3% in control versus 71.0% in *ob/ob*; *n *= 3 in each group). (C) LOX family gene expression in the peritoneal macrophages derived from 10∼12 week‐old male mice (*n *= 3 in each group). mRNA levels are expressed relative to average expression of *Lox* in peritoneal macrophages of WT mice. (D) Immunoblot analysis of pro‐LOX and LOX; and (E) LOX enzymatic activity in the cell lysates and culture medium of peritoneal macrophages derived from 10∼12 week‐old male mice (*n *= 3 in each group). (F) E_eff_ of the collagen gel on which the peritoneal macrophages derived from WT and *ob/ob* male mice were cultivated. Sample numbers and measurements taken are shown in Table S1. (G) Expression of *LOX* in PBMCs of obese subjects before (Pre) and 6‐months (*n *= 4)/12‐months (*n *= 5) after weight loss surgery. *P < 0.05 and **P < 0.01 by Student's *t*‐test in (C and E) and by Wilcoxon signed‐rank test in (F). Blue color in (G) indicates the results from humans

Because LOX regulation by different cell types among distinct tissues was reported,^7^ we herein tested various cell types and stimuli to delineate the cause of LOX upregulation in obese AT (Figure S2A–F). Macrophage RAW 264.7 cells exhibited significant *LOX* induction by lipopolysaccharide (LPS; inflammation) but not by CoCl_2_ (hypoxia) or TGFβ (fibrosis) (Figure 3A). LPS or a combined inflammatory regimen increased pro‐LOX and LOX proteins in RAW 264.7 cells but not the other cell types (Figure 3B and S2G and H). Importantly, LPS induced LOX enzymatic activity in RAW 264.7 cells (Figure 3C) and their ability to stiffen collagen gel; however, a LOX inhibitor, β‐aminopropionitrile (BAPN) abolished this ability (Figure 3D). In mouse primary peritoneal macrophages, LPS and cytokine cocktail induced pro‐LOX and LOX proteins and their enzymatic activity as well as the ability of macrophages to stiffen collagen gel (Figure S3). Collectively, these results implied increased LOX levels in macrophages caused by inflammatory stimuli resulted in AT stiffening. Thus, we hypothesized that obesity‐associated inflammatory responses upregulate LOX and collagen crosslinking, leading to AT stiffening and dysfunction.

**FIGURE 3:**
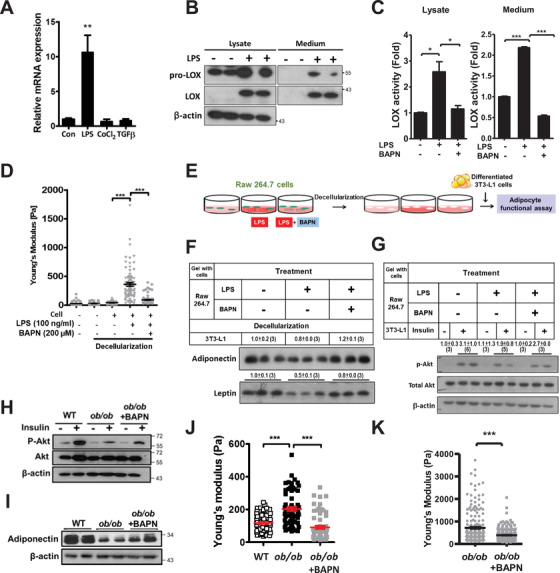
Macrophage LOX elevates substratum stiffness, consequently deteriorating functions of adipocytes cultivated on it. (A) Expression of *Lox* in RAW 264.7 cells treated with LPS (100 ng/ml), CoCl_2_ (20 nM) and TGFβ (20 ng/ml) for 24 h (n = 3 each). (B) Immunoblot analysis of pro‐LOX and LOX in the cell lysate and medium of RAW 264.7 cells in response to LPS (100 ng/ml). (C) LOX enzymatic activity in the cell lysate and medium of RAW 264.7 cells treated with LPS (100 ng/ml) and BAPN (200 μM) for 24 hr (n = 3 each). (D) E_eff_ of decellularized collagen gel after culturing with RAW 264.7 cells with or without LPS (100 ng/mL) and BAPN (200 μM) for 24hr. At least 60 distinct points from one to two plates were indented in each group. (E) The experimental design of cultivating differentiated 3T3‐L1 cells on RAW 264.7‐processed collagen gel. Immunoblot analysis of (F) adipokine secreted in culture medium and (G) insulin‐induced Akt phosphorylation (Ser473) in cell lysate of adipocytes cultivated on RAW 264.7‐processed collagen gel, of which RAW 264.7 were treated with LPS (100 ng/mL) and BAPN (200 μM) for 24 hr. The intensities of bands, quantified densitometrically relative to the control, are shown with the sample number in parentheses. (H) Immunoblot analysis of Akt Ser473 phosphorylation after 4‐h 100 nM insulin stimulation in AT explants of male WT and *ob/ob* mice with overnight 200 μM BAPN treatment. (I) Immunoblot analysis of adiponectin in AT explants. (J) E_eff_ of AT explants from 8–12 week‐old male WT and *ob/ob* mice with or without 200 μM BAPN treatment for 24 h. At least 60 distinct points from one to two mice were indented in each group. (K) E_eff_ in the gonadal AT of 6 to 8‐week‐old male *ob/ob* (from 3 mice in each group) treated with 600 mg/kg/day BAPN for 2 weeks. *P < 0.05, **P < 0.01, ***P < 0.001 by one‐way ANOVA with Tukey HSD test in (A and C) and by Wilcoxon signed‐rank test in (D, J, and K). Sample numbers and measurements taken in (D, J, and K) are shown in Table S1

Previous studies demonstrated that stiffened environment jeopardizes adipogenesis and mature adipocyte functions.^8^, ^9^ Based on the culture system and results established in Figure 3D, providing a bidirectional tool studying not only cell‐to‐matrix but also matrix‐to‐cell communications, we then tested whether LOX‐stiffened environment mediated by inflamed macrophages changed adipocyte behavior. RAW 264.7 cells were firstly cultivated on collagen gel with LPS and BAPN. After a decellularization procedure of washing away RAW 264.7 cells and residual chemicals, differentiated 3T3‐L1 adipocytes were then seeded on the macrophage‐processed collagen gel (Figure 3E). Adipokine secretion and insulin sensitivity diminished in adipocytes seeded on LPS‐treated macrophage‐processed gel, but BAPN co‐treatment during LPS administration restored these reductions (Figure 3F and G). These results implied that LOX, produced by inflamed macrophages, mediates substratum stiffness and subsequently affects adipocyte function.

The involvement of LOX and macrophage in adipose tissue stiffening and metabolism was dissected with LOX inhibition by BAPN and macrophage depletion by clodronate. BAPN has shown a beneficial effect on glucose metabolism.^4^, ^10^ *Ex vivo* BAPN treatment significantly enhanced insulin‐induced Akt phosphorylation and adiponectin expression, accompanied by decreased *ob/ob* AT stiffness (Figure 3H–J and S4). Consistently, in vivo BAPN treatment in *ob/ob* mice reduced AT stiffness (Figure 3K). In vivo clodronate treatment reduced pro‐LOX and LOX proteins, LOX enzymatic activity, and E_eff_ in *ob/ob* gonadal AT (Figure 4A–C) on top of the overall improvements in metabolism previously reported.

**FIGURE 4:**
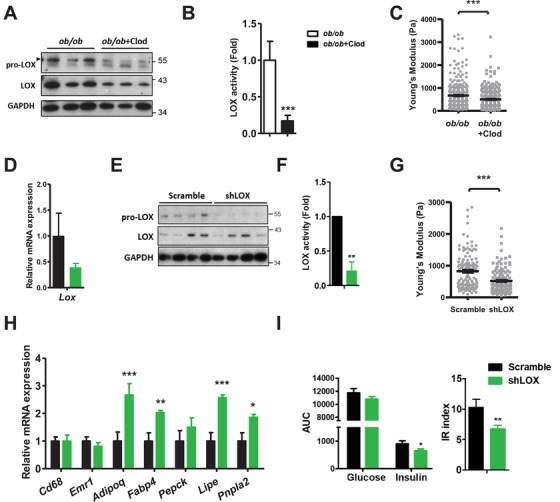
*ob/ob* mice treated with clodronate or reconstituted with LOX‐knockdowned BMCs exhibit lower AT stiffness and better glucose metabolism. (A) Immunoblot analysis for pro‐LOX and LOX, (B) LOX enzymatic activity, (C) E_eff_ of gonadal AT in 10∼12 week‐old male *ob/ob* mice treated with vehicle (*n *= 3) or clodronate (*n *= 5). Clodronate and PBS liposome were intraperitoneally injected with the dosage of 40 mg/kg for the first injection and 10 mg/kg for subsequent injections every 3 days for 6 weeks. (D) Expression of *Lox* (*n *= 3 in scramble and *n *= 5 in shLOX) and (E) immunoblot analysis for pro‐LOX and LOX in the gonadal AT of 10–12 week‐old *ob/ob* mice reconstituted with lentivirus transduced scramble or LOX‐knockdowned (shLOX) BMCs. Recipient male and female *ob/ob* mice were subjected to 7 Gy of irradiation one day before transplantation, of which 10^6^ transfected donor BMCs were delivered through tail vein injection. (F) LOX enzymatic activity, (G) E_eff_, and (H) expression of genes for macrophage markers (*Emr1* and *Cd68)* and adipocyte functional signatures in the gonadal AT of *ob/ob* mice reconstituted with scramble or shLOX BMCs (*n *= 3 in each group). (I) Plasma glucose and insulin AUC and IR index during OGTT in *ob/ob* mice reconstituted with scramble (*n *= 17) or shLOX (*n *= 20) BMCs 5∼8 weeks after BMTP. *P < 0.05, **P < 0.01, ***P < 0.001 by Student's *t*‐test in (B, D, F, H, and I) and by Wilcoxon signed‐rank test in (C) and (G). Sample numbers and measurements taken in (C) and (G) are shown in Table S1

To further assess the involvement of macrophage LOX in AT stiffening and systemic metabolism in vivo, we adopted bone marrow transplantation to reconstitute LOX‐knockdown bone marrow cells (BMCs, Figure S5A) in *ob/ob* mice. Eight weeks after transplantation, *ob/ob* mice receiving LOX‐knockdown BMCs exhibited diminished *Lox* transcript and pro‐LOX protein levels in gonadal AT (Figure 4D and E). Transplantation markedly suppressed LOX activity and reduced AT stiffness (Figure 4F and G). LOX knockdown in BMCs did not affect macrophage infiltration into AT, reflected by persisting macrophage markers, but improved AT functioning manifested by increased expression of adipocyte functional signature genes in *ob/ob* AT (Figure 4H). While LOX knockdown in BMCs had no impact on the body or AT weight (Figure S5B), a reduction in insulin area under curve and insulin resistance index during oral glucose tolerance test was discovered after transplantation (Figure 4I). These results suggested the involvement of macrophage LOX in obesity‐induced AT stiffening and metabolic malfunction.

In conclusion, we identified inflamed macrophages as key players in substratum stiffening by upregulating and releasing LOX, which further stiffened the environment where the cells were cultivated, leading to dysfunctional adipocytes. Macrophage depletion or LOX inhibition attenuated obesity‐induced LOX and AT stiffening. Furthermore, *ob/ob* mice reconstituted with LOX‐knockdown BMCs showed decreased AT stiffness and improved metabolism. Ongoing studies have gradually untangled underlying mechanisms of obesity, our study at least supports LOX derived from inflamed macrophages exerts AT stiffening and malfunction in obesity. This research also extends our perception of well‐known chemical effects of macrophages to a newly explored mechanical impact in obesity.

## CONFLICT OF INTEREST

The authors declare no conflict of interest.

## Supporting information

Supporting Information
