# Supplementary material for: Inflammation‐induced macrophage lysyl oxidase in adipose stiffening and dysfunction in obesity
Source: Clin Transl Med. 2021 Sep 16;11(9):e543. doi: 10.1002/ctm2.543 (PMC8444557; doi:10.1002/ctm2.543)
Supplement: Supplementary file 1 — Supporting Information [file CTM2-11-e543-s001.pdf]

Supplemental materials

METHODS

Human and AT procurement

Patients who attended the Weight Control Clinic in National Cheng Kung University Hospital (NCKUH, Tainan City, Taiwan) for bariatric surgery (mini-gastric bypass, sleeve gastrectomy, and gastric banding) (BMI $\geq$ 35 kg/m^2) was included in our study. Information of subjects included were provided in Table S2. Obese human subcutaneous AT (1~2 g) was obtained during the procedure of bariatric surgery. Six months after bariatric surgery, subcutaneous AT (1~2 g) was obtained from the paraumbilical region by needle aspiration under local anesthesia. Lean human subcutaneous AT (1~2 g) was collected during regular liposuction procedure, and harvested from periumbilical region, which was regular surgical incision wound of liposuction. After saline rinse, one piece was processed for the histology; the other piece was frozen in liquid nitrogen and stored at -80°C for subsequent RNA or protein analyses. Peripheral blood mononuclear cells (PBMCs) were isolated from obese subjects undergoing bariatric surgery by density gradient centrifugation using Ficoll-Paque™ PREMIUM (GE Healthcare, Pittsburgh, PA, USA) [1, 2]. All the informed consent, tissue collection procedure, clinical data acquisition and postoperative report of adverse effect were approved and regulated by the Institutional Review Board of National Cheng Kung University Hospital.

Histological analysis of AT

Fresh AT was fixed in 10% phosphate-buffered formalin overnight, and paraffin sections of 10 µm were processed for immunostaining. Collagen networks was visualized by PSR staining (Abcam, Cambridge, UK) in bright field; while collagen bundles was captured by the same staining using crossed polars (polarized). The abovementioned staining was counterstained with hematoxylin (Sigma-Aldrich, St. Louis, MO, USA). Immune cells infiltration and crown-like structure were determined by hematoxylin and eosin (H&E) staining.

SHG signal processing and quantification

Images of unstained sections were acquired using a Genesis system (HistoIndex Pte. Lte., Singapore) in which second harmonic generation (SHG) microscopy was used to visualize collagen and two-photon excited fluorescence (TPEF) microscopy was utilized for visualization of the other cell structures. The samples were laser-excited at 780 nm, SHG signals were recorded at 390 nm, and TPEF signals were recorded at 550 nm. Extracted SHG signals were identified from original SHG images by applying connected domain algorithm. The collagen binary image is generated from the SHG signal using the Otsu’s automatic threshold method. Each connected component in the collagen binary image is a collagen string. A thinning algorithm was applied to collagen strings to identify the skeleton of each string. The intersections are defined as the branch points of the skeleton. Seventeen randomly selected views were used for quantification in Figure 1B. SHG percentage (% of SHG) is defined as the ratio of SHG area to overall tissue area. Number

of cross-links (intersection) is calculated as the total number of the intersections normalized by overall tissue area.

AFM cantilevers and measurement parameters

The elastic modulus of the tissues and gels was measured using atomic force microscopy (AFM, NanoWizard® II system, JPK, Berlin, German) indentation, followed by previous studies with some modifications [3, 4]. In short, to assess the effective Young's modulus (E_{eff}) of tissues and gels obtained by AFM, a 5 μm (in diameter) polystyrene bead-modified tip-less cantilever (ARROW-TL1-50, NanoWorld, Neuchâtel, Switzerland) was utilized. The nominal spring constants of all cantilevers ranged from 0.02 to 0.08 N/m. Thermo noise method is used to calibrate each cantilever prior to each experiment. The applied forces in this study were set to 1 nN and the corresponding displacements on cells were recorded simultaneously. The approaching and retracting rates of cantilever were set at 1 μm/sec. The on-site temperature was 31℃ during whole experimental process. Tissue samples were cut from the AT and constraint by glued coverslips in 6 cm in diameter plastic dish which was filled with PBS. Tissue thickness is more than 30 μm in this study, therefore the limit of indentation depth was set under 3 μm, The Young's modulus of AT was calculated based on Hertzian contact model. Due to the loading rate applied in this study, the tissue behavior is predominantly elastic. Thus, the Poisson's ratio is set as 0.5 to fit the elastic material assumptions. At least 60 distinct points with 20 um span were indented in one sample. Sample numbers and measurements taken for each AFM data are shown in Table S1.

Cells and treatments

RAW 264.7 and 3T3-L1 cell lines were obtained from Food Industry Research and Development Institute, Taiwan. Over-confluent 3T3-L1 cells were incubated with the medium containing insulin (1.7 μM), dexamethasone (1 μM) and 1-methyl-3-isobutyl-xanthine (0.5 M) for 72 hrs. Differentiating 3T3-L1 cells were then changed to the medium containing DMEM with 10% fetal bovine serum and insulin (1.7 μM) for 6 days with medium changed on day 3. Oil-Red O staining was employed to visualize oil droplet in fixed 9-day differentiated adipocytes as described [5]. For insulin stimulation, differentiated 3T3-L1 cells were starved in DMEM for 16 hrs prior to 100 nM insulin stimulation for 5 mins. For lipolytic stimulation, differentiated 3T3-L1 cells were exposed to 50 μM isoproterenol prepared in KRBHA buffer for 4 hrs. For transferring differentiated 3T3-L1 cells from plastic dish onto collagen gel, cells were first trypsinized for 3 mins and centrifuged with 100 g for 5 mins; after removing the supernatant, the cells were gently pipetted with culture medium and equally distributed onto collagen gels with different rigidities. All the subsequent treatments were take place after an overnight sit [6]. For inflammatory treatments, 100 ng/mL LPS for 24 hrs or the combination of TNF-α (50 ng/ml) and INF-γ (100 ng/ml) for 24 hrs were used. For BAPN treatment, 200 μM BPAN for 24 hrs were used for LOX inhibition.

Collagen gel preparation and decellularization

Collagen gels were made of 1 mg/mL type I collagen (BD Bioseince, San Jose, CA, USA) dissolved in 5.7X DMEM, 0.1 M HEPES, 0.17 M CaCl2, 2.5% NaHCO3 and adjusted to pH=7.4 with 1 N NaOH as described [7]. 0.1% glutaraldehyde (GLA) was used to chemically

cross-linked collagen to create a stiffer gel as described in Figure S4. In order to eliminate toxicity of GLA residing in collagen gel, the gel was rinsed several times with refreshed NH_4Cl (50 mM) in PBS with agitation for 24 hrs. Pre-differentiated or day-9 post-differentiated 3T3-L1 cells, as well as Raw 264.7 cells, were then cultivated on collagen gels for subsequent stimulations as indicated in Figure S4 and 3. In Figure 3, decellularization was done with 2 mM EDTA in sterilized ddH2O added on collagen gel with agitation for 2 days to remove Raw 267.4 cells.

Animals

Leptin-deficient (*ob/ob*) mice and control littermates, as well as C57BL/6 mice, obtained from National Laboratory Animal Center, Taiwan were fed regular chow. BAPN (600 mg/kg/day, Sigma-Aldrich, St. Louis, MO, USA) was intraperitoneally given for 2 weeks. Clod and PBS liposome (Liposoma, Amsterdam, Netherland) were intraperitoneally injected with the dosage of 40 mg/kg for the first injection and 10 mg/kg for subsequent injections every 3 days for 6 weeks. Animals were kept in a specific-pathogen-free barrier facility and handled in accordance with procedures approved by the Institutional Animal Care and Use Committees of National Cheng Kung University. For isolation of mouse peritoneal macrophages, WT or *ob/ob* mice were intraperitoneally injected with 2 mL of 3% sterile thioglycollate 3 days prior to euthanasia. Cells were collected by lavage of the peritoneal cavity with 5 mL of sterile cold 1X PBS, followed with incubation of red blood cell (RBC) lysis buffer to remove RBCs. Cells were centrifuged at 200x g for 5 mins and re-suspended in RPMI.

Immunofluorescence staining and image analysis

Paraffin-section containing AT sections of 10 μm were deparaffinized, rehydrated, and boiled for epitope retrieval using an antigen retrieval buffer at pH = 6.0 in peroxidase-free water (Opal 4-color IHC Kit, PerkinElmer, Waltham, MA). Tissue sections were blocked (Thermo SuperBlock™ Blocking Buffer with 3% BSA) for 60 mins at 37 ℃ and incubated with primary antibodies overnight at 4℃. Primary antibodies include: LOX (dilution 1:1000; ab31238, Abcam, Cambridge, UK), F4/80 (dilution 1:1000; ab6640, Abcam) and ATGL (dilution 1:8000; 2138, Cell Signaling, Boston, MA, USA). The sections were washed with TBST and incubated with Polymer HRP-conjugated secondary antibodies for 60 mins at room temperature. Secondary antibodies include: Anti-Rabbit IgG HRP 1:500 (18-8816-33, Rockland, Limerick, PA, USA) and anti-Rat IgG HRP 1:300 (ab6845, Abcam). The slides were then incubated at room temperature for 10 min with one of the following Alexa Fluor tyramides (Opal 520, Opal 570 and Opal 690 with 1:100 dilution; PerkinElmer). The primary antibodies were stained with repeat procedures including antigen stripping and blocking steps. After three additional washes, the slides were mounted by the mounting media containing fluoroshield with DAPI. The images were visualized by confocal microscopy (TE-2000 Laser Scanning Confocal Microscope, Nikon, Melville, NY, USA) and analyzed by TissueQuest (TissueGnostics GmbH, Vienna, Austria) [8, 9]. For the scattering plot of LOX and F4/80 in Fig. 2B, the TissueQuest software identified LOX and F4/80-positive cells using an algorithm that first identified DAPI-labelled nuclei and then measured total fluorescence intensity of each channel around individual nucleus. Only cells exceeding the basal fluorescence intensity in the respective channel were accepted as marker-positive cells.

RNA analysis

RNA from gel-seeded cells or tissues was extracted using the REzol reagent (Protech Technology Enterprise, Taipei, Taiwan), and followed by chloroform extraction. mRNA levels were analyzed with real-time quantitative RT-PCR, using β-actin or Gapdh as reference gene in each reaction. Sequences of the primers used for RT-PCR assays are shown in Table S3.

Immunoblot analysis

Ten to fifteen micrograms of total proteins were subjected to electrophoresis, transferred to PVDF membranes, and probed with antibodies listed in Table S4. Immunoreactive proteins were detected using an enhanced chemiluminescence Western blotting detection system (GE Healthcare, Pittsburgh, PA, USA).

Glucose metabolic assays

Mice were fasted for 4 hrs and given an oral glucose bolus (2 g/kg body weight). Blood samples were collected before and at 15, 30, 60, and 120 mins after glucose was given. Plasma glucose concentration was determined by a glucose colorimetric test (Autokit Glucose, Wako, Richmond, VA, USA). Insulin was measured using a mouse insulin ELISA (Ultrasensitive Mouse Insulin ELISA, Mercodia AB, Uppsala, Sweden). The IR index was calculated as the product of the areas under glucose and insulin curves (AUC) in glucose tolerance tests as previously described [10]. Homeostatic model assessment (HOMA) index was calculated as the product of fasting glucose and insulin level divided by 22.5.

LOX enzymatic activity

Homogenized ATs and extracted macrophage cell lysate were incubated with the buffer containing the substrate 1,5-diaminopentane (Fluka, St. Louis, MO, USA). The fluorescence from HRP-coupled reactions triggered by hydrogen peroxide was measured using a fluorescence microplate reader with excitation and emission wavelengths at 538 and 590 nm. Parallel assays were prepared with 100 mM of added BAPN to completely inhibit the LOX activity, and the difference in fluorescence intensity was recorded. The LOX activity was calculated as the increase in fluorescence units over time above the BAPN controls divided by cell protein content or followed by normalization to controls in each experimental group [4]. Data are normalized to the average of its respective control group.

Culture of explanted AT

Gonadal fat pads were removed from mice, manually fragmented in size (0.2–0.3 cm^3) and washed by PBS. Explanted tissue were incubated in DMEM and treated with or without 200 μM BAPN for 24 hrs. Additional 1 hr insulin (1 μM) stimulation was given to explanted AT to assess insulin sensitivity and lipolysis inhibition [11].

Bone marrow transplantation

Eight-to-ten weeks old recipient male and female *ob/ob* mice were subjected to 7 Gy of irradiation from X-ray irradiator (Rad source RS2000, Buford, GA, USA) with 3 mm copper filter to induce bone marrow aplasia one day before transplantation. BMCs isolated from donor C57BL/6 mice were transduced with lentivirus (MOI=40) for 24 hrs. Lentivirus with scramble (TRCN 00000231719) or shLOX (TRCN 0000011848 and TRCN

0000011851) plasmids were obtained from the National RNAi Core Facility Platform (Academia Sinica, Taiwan). 10^6 cells were delivered to irradiated recipient mice through tail vein injection.

Statistical analysis

Values are presented as means ± SEM. Student's *t*-test or Wilcoxon signed-rank test was used for comparing two groups, and one-way-ANOVA was used for analyzing three groups followed by post hoc Tukey's least significant difference for comparisons. The statistics taken in each analysis was described in the figure legends. Statistically significance was set at $P<0.05$.

References

1. Fidan I, Yesilyurt E, Kalkanci A, et al. Immunomodulatory effects of voriconazole and caspofungin on human peripheral blood mononuclear cells stimulated by Candida albicans and Candida krusei. *The American journal of the medical sciences*. Sep 2014;348(3):219-23. doi:10.1097/maj.0000000000000236

2. Fink C, Gaudet JM, Fox MS, et al. 19F-perfluorocarbon-labeled human peripheral blood mononuclear cells can be detected in vivo using clinical MRI parameters in a therapeutic cell setting. *Scientific Reports*. 2018/01/12 2018;8(1):590. doi:10.1038/s41598-017-19031-0

3. Chiou Y-W, Lin H-K, Tang M-J, Lin H-H, Yeh M-L. The Influence of Physical and Physiological Cues on Atomic Force Microscopy-Based Cell Stiffness Assessment. *PloS one*. 2013;8(10):e77384. doi:10.1371/journal.pone.0077384

4. Chen J-Y, Tsai P-J, Tai H-C, et al. Increased Aortic Stiffness and Attenuated Lysyl Oxidase Activity in Obesity. *Arteriosclerosis, Thrombosis, and Vascular Biology*. 2013/04/01 2013;33(4):839-846. doi:10.1161/ATVBAHA.112.300036

5. Xu H, Barnes GT, Yang Q, et al. Chronic inflammation in fat plays a crucial role in the development of obesity-related insulin resistance. *J Clin Invest*. Dec 2003;112(12):1821-30. doi:10.1172/jci19451

6. Kilroy G, Burk DH, Floyd ZE. High efficiency lipid-based siRNA transfection of adipocytes in suspension. *PloS one*. 2009;4(9):e6940-e6940. doi:10.1371/journal.pone.0006940

7. Wei WC, Hsu YC, Chiu WT, et al. Low substratum rigidity of collagen gel promotes ERK phosphorylation via lipid raft to augment cell migration. *Journal of cellular biochemistry*. Mar 1 2008;103(4):1111-24. doi:10.1002/jcb.21482

8. Ecker RC, Steiner GE. Microscopy-based multicolor tissue cytometry at the single-cell level. *Cytometry Part A*. 2004;59A(2):182-190. doi:10.1002/cyto.a.20052

9. Steiner GE, Ecker RC, Kramer G, Stockenhuber F, Marberger MJ. Automated data acquisition by confocal laser scanning microscopy and image analysis of triple stained immunofluorescent leukocytes in tissue. *Journal of immunological methods*. Apr 03 2000;237(1-2):39-50.

10. Tsai Y-S, Tsai P-J, Jiang M-J, et al. Decreased PPARγ Expression Compromises Perigonadal-Specific Fat Deposition and Insulin Sensitivity. *Molecular Endocrinology*. 2009;23(11):1787-1798. doi:10.1210/me.2009-0073

11. Tsai YS, Kim HJ, Takahashi N, et al. Hypertension and abnormal fat distribution but not insulin resistance in mice with P465L PPARgamma. *J Clin Invest*. Jul 2004;114(2):240-9. doi:10.1172/JCI20964 [doi]

Figure S1

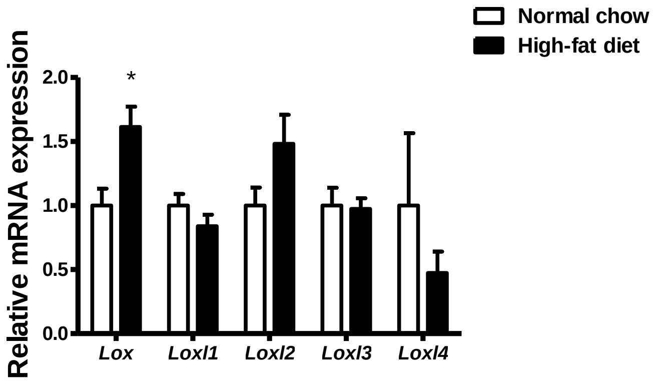

Figure S1. Obesity is associated with increased LOX family gene expression in the AT of high-fat diet induced obese mice.

LOX family gene expression in gonadal AT of 6-month-old male mice fed normal chow and a high-fat diet (D12492, Research Diets) for 3 months. mRNA levels (n=5 each) are expressed relative to average expression in normal chow mice. *P<0.05 by Student's t-test.

Figure S2

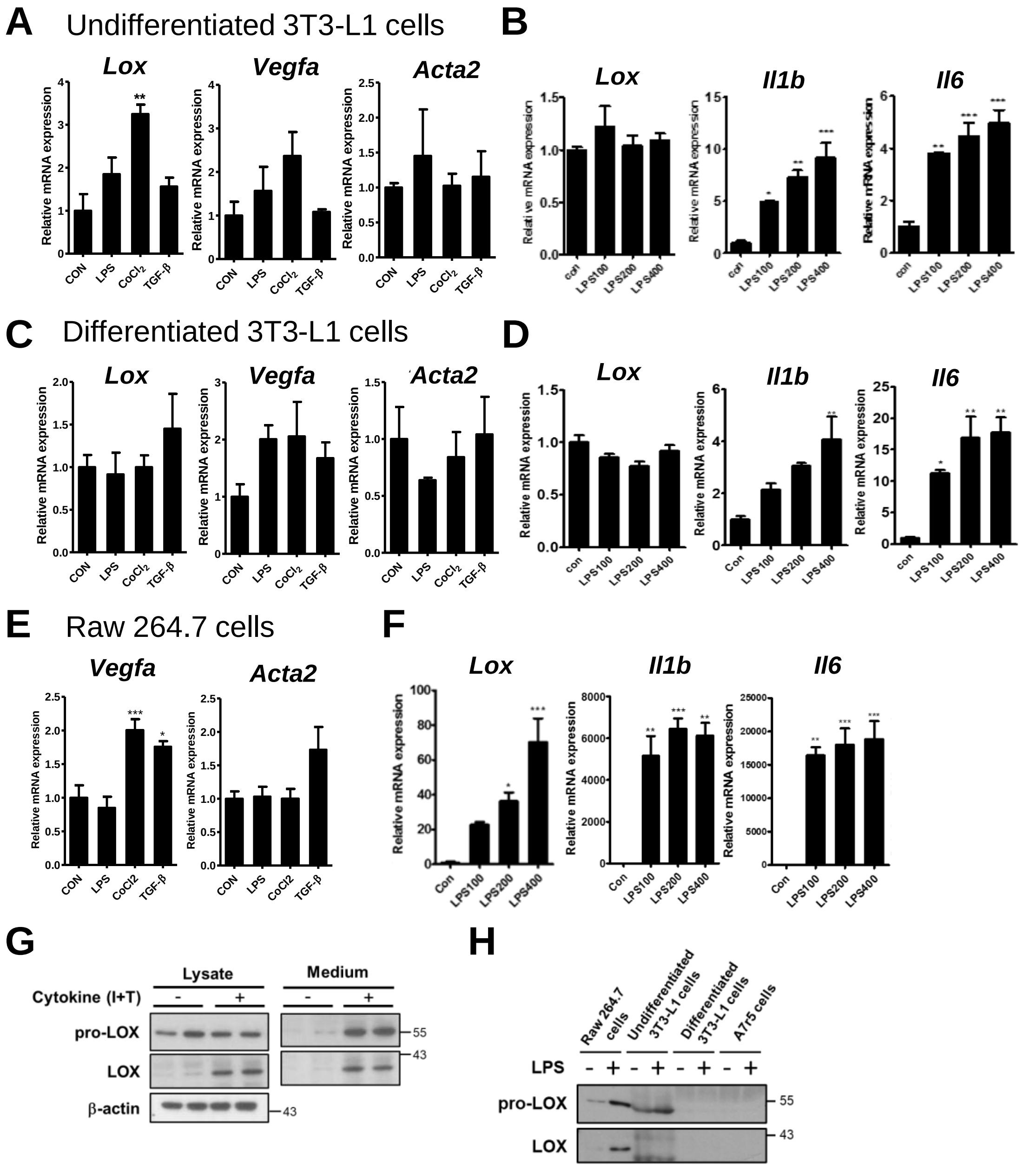

A
Undifferentiated 3T3-L1 cells
B
C
Differentiated 3T3-L1 cells
D
E
Raw 264.7 cells
F
G
H
Lox
Vegfa
Acta2
Il1b
Il6
Relative mRNA expression
CON
LPS
CoCl2
TGF-β
con
LPS100
LPS200
LPS400
Con
Lysate
Medium
Cytokine (I+T)
pro-LOX
LOX
β-actin
55
43
Raw 264.7 cells
Undifferentiated 3T3-L1 cells
Differentiated 3T3-L1 cells
A7r5 cells
LPS

Figure S2. Undifferentiated and differentiated 3T3-L1 cells do not respond to inflammatory (LPS) or fibrotic (TGFβ) stimuli for LOX induction.

(A) Expression of *Lox*, *Vegfa*, and *Acta2* in undifferentiated 3T3-L1 cells treated with LPS (100 ng/ml), $CoCl_2$ (20 nM), and TGFβ (20 ng/ml) for 24 hr (*n*=3 each). **(B)** Expression of *Lox*, *Il1b*, and *Il6* in undifferentiated 3T3-L1 cells treated with LPS from 100 to 400 ng/ml (*n*=3 each). **(C)** Expression of *Vegfa* and *Acta2* in differentiated 3T3-L1 cells treated with LPS (100 ng/ml), $CoCl_2$ (20 nM), and TGFβ (20 ng/ml) for 24 hr (*n*=3 each). **(D)** Expression of *Lox*, *Il1b*, and *Il6* in differentiated 3T3-L1 cells treated with LPS from 100 to 400 ng/ml (*n*=3 each). **(E)** Expression of *Vegfa* and *Acta2* in RAW 264.7 cells treated with LPS (100 ng/ml), $CoCl_2$ (20 nM), and TGFβ (20 ng/ml) for 24 hr (*n*=3~4). **(F)** Expression of *Lox*, *Il1b*, and *Il6* in RAW 264.7 cells treated with LPS from 100 to 400 ng/ml (*n*=3~4). **(G)** Immunoblot analysis of pro-LOX and LOX in the cell lysate and culture medium of RAW 267.4 cells treated with a combination of TNF-α (50 ng/ml) and INF-γ (100 ng/ml) (Cytokine I+T) for 24 hrs. **(H)** Immunoblot analysis of pro-LOX and LOX in the culture medium of RAW 264.7, undifferentiated 3T3-L1, differentiated 3T3-L1, and A7r5 vascular smooth muscle cells in response to LPS (100 ng/ml). *$P<0.05$, **$P<0.01$, ***$P<0.001$ by one-way ANOVA with Tukey HSD test.

Figure S3

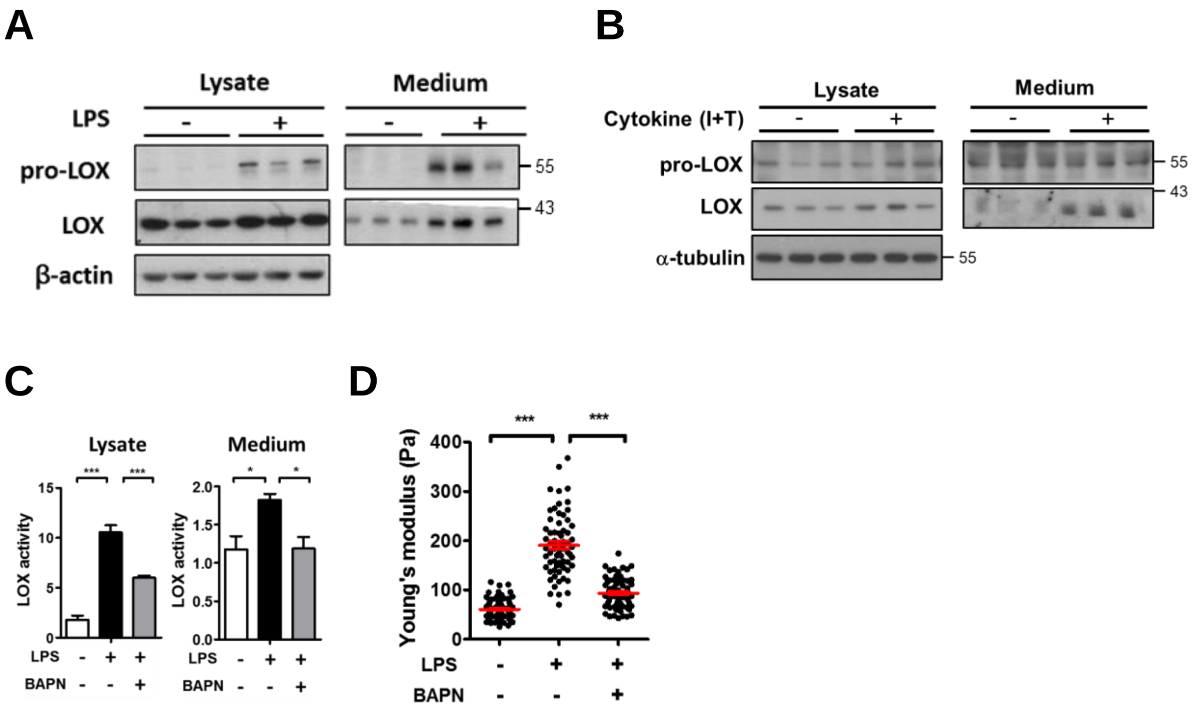

Figure S3. LPS induced LOX in peritoneal macrophages of WT mice.
Immunoblot analysis of pro-LOX and LOX in the cell lysate and culture medium of peritoneal macrophages treated with **(A)** LPS (100 ng/ml) or **(B)** a combination of TNF-α (50 ng/ml) and INF-γ (100 ng/ml) (Cytokine I+T) for 24 hrs. **(C)** LOX enzymatic activity in the cell lysate and culture medium of peritoneal macrophages treated with LPS (100 ng/ml) and BAPN (200 μM) (n=3). **(D)** E_{eff} of decellularized collagen gel after culturing with peritoneal macrophages of WT mice. *P<0.05, ***P<0.001 by one-way ANOVA with Tukey HSD test.

Figure S4

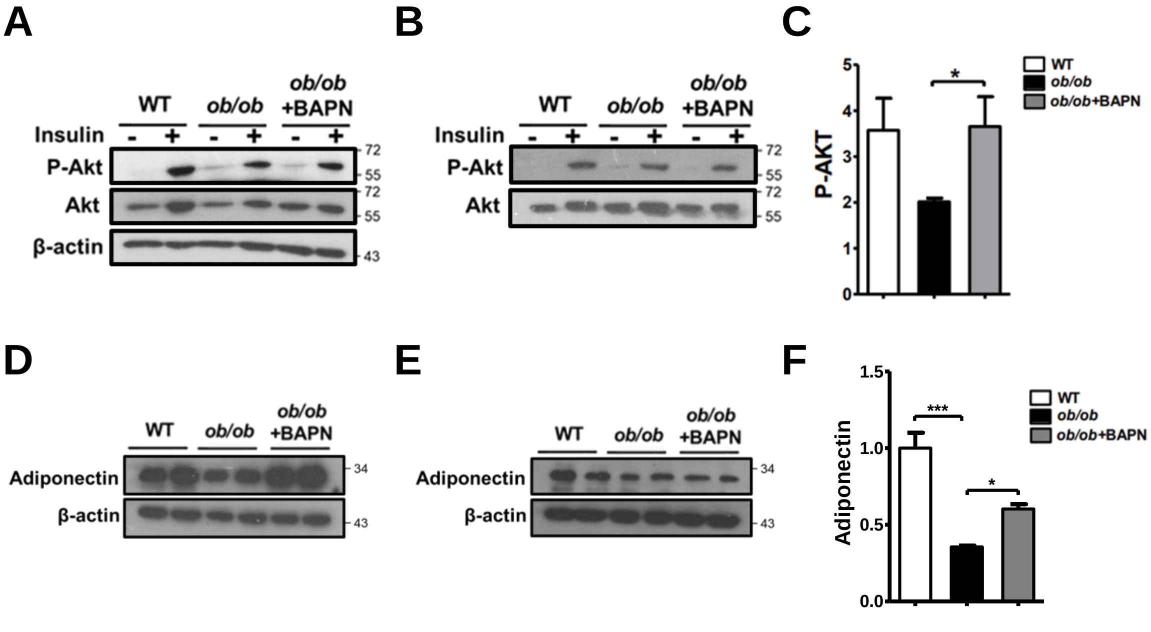

Figure S4. ***Ex vivo*** **LOX inhibition reverses adipocyte dysfunction.** Immunoblot analysis **(A and B)** and quantification (n=4 each) **(C)** of Akt Ser473 phosphorylation after 4-hr 100 nM insulin stimulation in AT explants of WT and *ob/ob* mice with overnight 200 μM BAPN treatment. Immunoblot analysis **(D and E)** and quantification (n=4 each) **(F)** of adiponectin in AT explants. *$P<0.05$ and ***$P<0.001$ by one way ANOVA with Tukey HSD test.

Figure S5

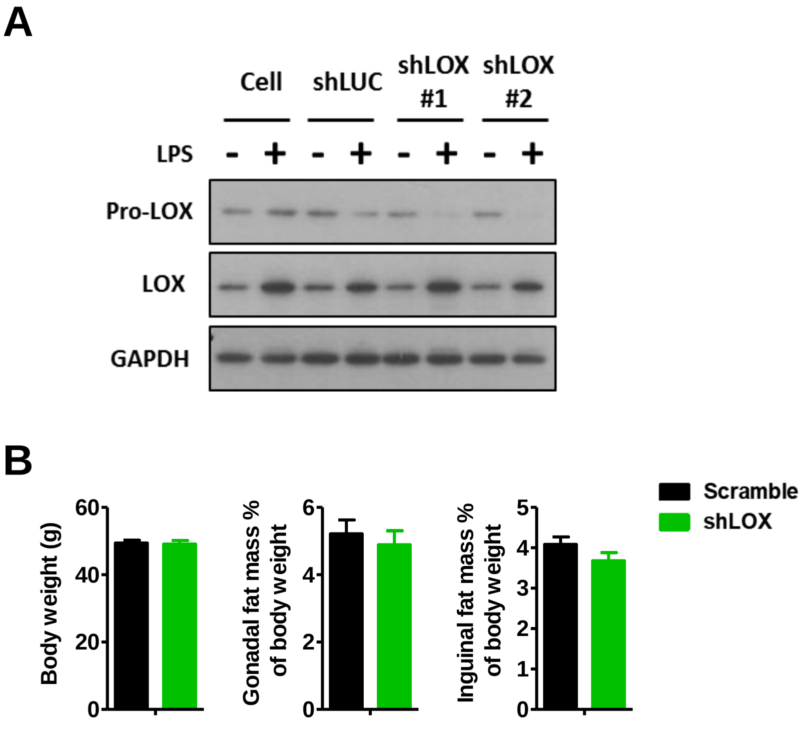

Figure S5. *ob/ob* mice reconstituted with LOX-knockdowned BMCs exhibit lower collagen deposition.

(A) Immunoblot analysis for pro-LOX, LOX in RAW 264.7 cells transfected with LOX knockdown plasmids. **(B)** Body weight and gonadal/inguinal fat mass in percentage of body weight 10~16 weeks after BMTP in *ob/ob* mice reconstituted with scramble (*n*=17) or shLOX (*n*=18).

Table S1. List of *n* value and measurement taken in each AFM data

Figure	Group	*n* value	Measurements
1F	WT	2	65, 63
	ob/ob	2	69, 62
1G	Lean	3	65, 67, 66
	Obese	3	61, 65, 64
1I	Pre	3	61, 63, 60
	Post	3	63, 67, 59
2F	WT	2	66, 66
	ob/ob	2	65, 64
3D	Lane 1	1	84
	Lane 2	1	69
	Lane 3	2	65, 74
	Lane 4	2	65, 66
	Lane 5	2	70, 71
3J	WT	2	71, 67
	ob/ob	2	61, 70
	ob/ob+BAPN	2	61, 63
3K	*ob/ob*	3	87, 63, 64
	ob/ob+BAPN	3	67, 70, 95
4C	*ob/ob*	3	71, 69, 89
	ob/ob+Clod	5	73, 89, 75, 79, 68
4G	Scarmble	2	65, 65
	shLOX	2	63, 66

Table S2. List of Human Samples

S/N	Gende r	Age	Procedure type (Mini-gastric bypass, Sleeve gastrectomy, or Gastric banding)	BMI (Before Surgery)	BMI (6mth After Surgery)	BMI (12mth After Surgery)
01	F	36	Sleeve gastrectomy	47.0	37.0	N/A
02	M	35	Mini-gastric bypass	70.7	47.3	N/A
03	F	32	Mini-gastric bypass	41.3	30.4	N/A
04	M	27	Sleeve gastrectomy	40.8	25.4	N/A
05	M	36	Sleeve gastrectomy	48.3	35.8	N/A
06	M	51	Sleeve gastrectomy	56.4	44.1	N/A
07	M	23	Sleeve gastrectomy	47.1	38.2	N/A
08	M	48	Mini-gastric bypass	33.5	27.6	N/A
09	F	23	Sleeve gastrectomy	43.2	N/A	30.9
10	F	29	Sleeve gastrectomy	42.6	31.4	31.4
11	F	44	Mini-gastric bypass	48.2	35.5	33.9
12	F	35	Sleeve gastrectomy	44.1	32	N/A
13	F	29	Gastric banding	38.9	32.4	31.8
14	M	29	Sleeve gastrectomy	38.5	27.2	N/A
15	F	37	Sleeve gastrectomy	48.1	34.5	34.4
16	F	41	Flank liposuction	18.5	N/A	N/A
17	M	64	Abdomen liposuction	22.6	N/A	N/A
18	F	23	Abdomen liposuction	16.0	N/A	N/A
19	M	26	Inner thigh liposuction	16.7	N/A	N/A
20	M	50	Abdomen liposuction	28.4	N/A	N/A
21	F	17	Abdomen liposuction	18.7	N/A	N/A
22	F	44	Abdomen liposuction	27.6	N/A	N/A

Table S3. List of Primers

Genes	Species	Forward primer	Reverse primer
Actb	Mouse	ACTGCCGCATCCTCTTCCTC	TGCCACAGG ATTCCATACCC
Gapdh	Mouse	TGACGTGCCGCCTGGAGAAA	AGTGTAGCCCAAGATGCCCTT
Emr1	Mouse	CTT GGCTATGGGCTTCCAGTC	GCAAGGAGGACAGAGTTTATCGTG
Il6	Mouse	AACGATGATGCACTTGCAGA	GAGCATTGGAAATTGGGGTA
Cd68	Mouse	AGCTGCCTGACAAGGGACACT	AGGAGGACCAGGCCAATGAT
Il1b	Mouse	GCAACTGTTCCTGAACTCAACT	ATCTTTTGGGGTCCGTCAAT
Adipoq	Mouse	ATCCTGGCCACAATGGCACA	CAAGAAGACCTGCATCTCCT
Pepck	Mouse	AGTCACCATCACCTCCTGGA	CAGAATCTCGAGTTGGGATG
Fabp4	Mouse	CACCGAGATTTCCTTCAAACT	GCCATCTAGGGTTATGATGC
Lipe	Mouse	TCT GCT GGC CCC TGA CA	AGA GCG CAA GCC ACA AGG T
Pnpla2	Mouse	AACACCAGCATCCAGTTCAA	GGTTCAGTAGGCCATTCCTC
Lox	Mouse	GTCACCAACATTACCACAGCA	CATAACATCCAGGACTCAATCC
Loxl1	Mouse	CTATGCCTGCACCTCTCACA	GTAGTTCCCAGGCTGCACAT
Loxl2	Mouse	TTTACGCTTCTCCTCCCAGA	TGAAGACTTCCATGCTGTGG
Loxl3	Mouse	GTGTCATGGGCATTACCACA	AAAGTTGGCGCACTCATACC
Loxl4	Mouse	AACTTTGGGGAACAGGGAGT	TCGCTGTCCATCATACTTGC
Vegfα	Mouse	TAGAGTACATCTTCAAGCCG	TCTTTCTTTGGTCTGCATTC
Acta2	Mouse	CGGCTTCGCTGGTGATGATG	TCCCTCTCTTGCTCTGGGCTT
LOX	Human	GATACGGCACTGGCTACTTCCA	GCCAGACAGTTTTCCTCCGCC
LOXL1	Human	GTCGCTACGTTTCTGCAACA	ATCCCTGTGGTTTTCATCCA
LOXL2	Human	CTGCCACATAGGTGGTTCCT	TGGCATTCGTTCAGACTCAG
LOXL3	Human	GTCCGGTGCAACCTACCTTA	TCCCAGAGTCCCAGTACCAG
GAPDH	Human	CACATGGCCTCCAAGGAGTAAG	CCAGCAGTGAGGGTCTCTCT

Table S4. List of Antibodies

Antibody	Brand, cat#
**Akt**	Cell Signaling, #9272
**p-Akt**	Cell Signaling, #4058
**β-actin**	Sigma, A5441
**a-tubulin**	Sigma, T5168
**GAPDH**	GeneTex, GTX627408
**pro-LOX**	Abcam, ab174316
**LOX**	Abcam, ab31238
**Adiponectin**	R&D, MAB1119
**Leptin**	Biovision, 5367-100
